# Supplementary material for: Systemic Inflammation and Oxidative Stress in Childhood Obesity: Sex Differences in Adiposity Indices and Cardiovascular Risk
Source: Biomedicines. 2024 Dec 29;13(1):58. doi: 10.3390/biomedicines13010058 (PMC11761629; doi:10.3390/biomedicines13010058)
Supplement: Supplementary file 1 [file biomedicines-13-00058-s001.zip › biomedicines-3371340-supplementary.pdf]

Results

Contingency Tables

Contingency Tables

	SBPcategory			
Gender	normal	hypertension	prehypertension	Total
1	32	10	2	44
2	20	14	2	36
Total	52	24	4	80

χ^2 Tests

	Value	df	p
χ^2	2.66	2	0.264
N	80		

Plots

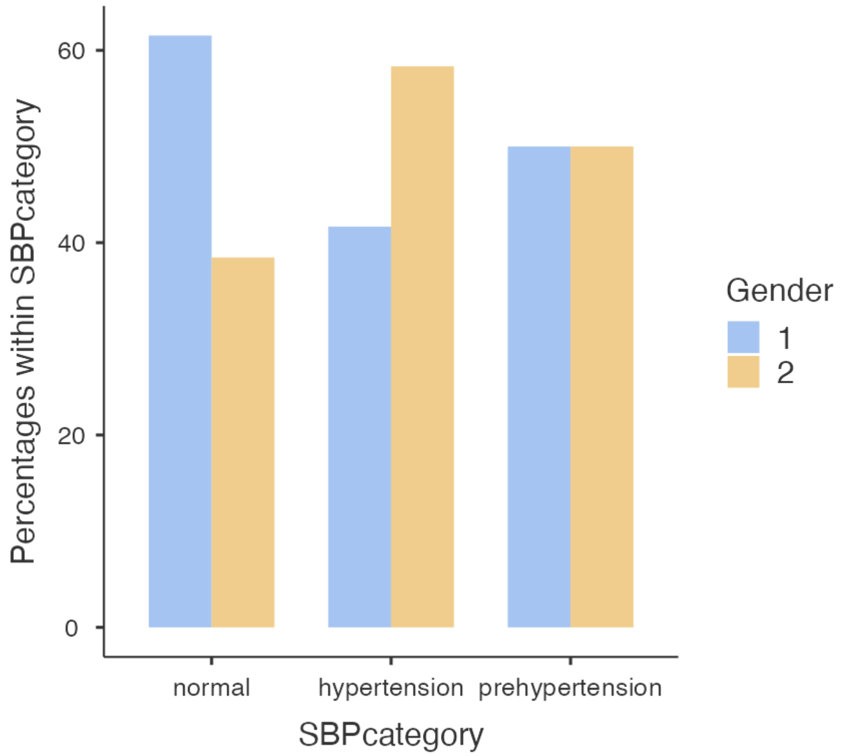

Contingency Tables

Contingency Tables

	DBPcategory			
Gender	normal	hypertension	prehypertension	Total
1	31	8	5	44
2	18	12	6	36
Total	49	20	11	80

χ² Tests

	Value	df	p
χ²	3.58	2	0.167
N	80		

Plots

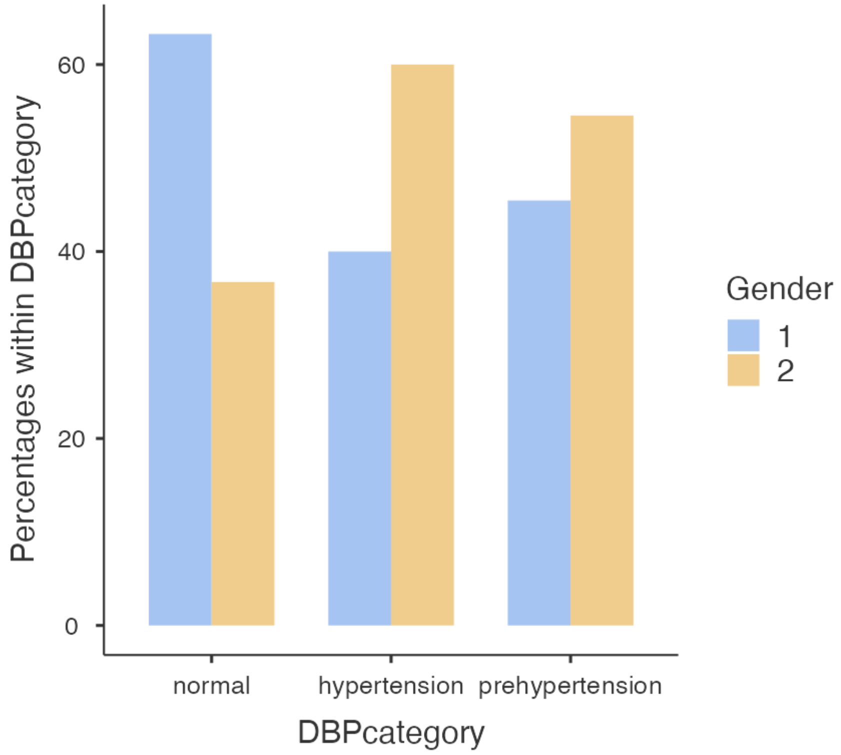

References

[1] The jamovi project (2024). *jamovi*. (Version 2.5) [Computer Software]. Retrieved from https://www.jamovi.org.

[2] R Core Team (2023). *R: A Language and environment for statistical computing.* (Version 4.3) [Computer software]. Retrieved from https://cran.r-project.org. (R packages retrieved from CRAN snapshot 2024-01-09).
